# Development and performance evaluation of a GIS-based metric to assess exposure to airborne pollutant emissions from industrial sources

**DOI:** 10.1186/s12940-019-0446-x

**Published:** 2019-01-25

**Authors:** Thomas Coudon, Aurélie Marcelle Nicole Danjou, Elodie Faure, Delphine Praud, Gianluca Severi, Francesca Romana Mancini, Pietro Salizzoni, Béatrice Fervers

**Affiliations:** 1 Département Cancer & Environnement, Centre Léon Bérard, 69008 Lyon, France; 2 Université Claude Bernard Lyon 1, 69100 Villeurbanne, France; 3 INSERM 1052, CNRS 5286, Centre de Recherche en Cancérologie de Lyon, 69373 Lyon, France; 4 Centre de Recherche en Épidémiologie et Santé des Populations (CESP, Inserm U1018), Facultés de Médecine, Université Paris-Saclay, UPS, UVSQ, Gustave Roussy, Villejuif, France; 5 Laboratoire de Mécanique des Fluides et d’Acoustique, UMR CNRS 5509, University of Lyon, Ecole Centrale de Lyon, INSA Lyon, Université Claude Bernard Lyon I, 36, avenue Guy de Collongue, 69134 Ecully, France

**Keywords:** Dioxins, Cadmium, Exposure assessment, GIS, Dispersion modelling, SIRANE, Cancer

## Abstract

**Background:**

Dioxins are environmental and persistent organic carcinogens with endocrine disrupting properties. A positive association with several cancers, including risk of breast cancer has been suggested.

**Objectives:**

This study aimed to develop and assess performances of an exposure metric based on a Geographic Information System (GIS) through comparison with a validated dispersion model to estimate historical industrial dioxin exposure for its use in a case-control study nested within a prospective cohort.

**Methods:**

Industrial dioxin sources were inventoried over the whole French territory (*n* > 2500) and annual average releases were estimated between 1990 and 2008. In three selected areas (rural, urban and urban-costal), dioxin dispersion was modelled using SIRANE, an urban Gaussian model and exposure of the French E3N cohort participants was estimated. The GIS-based metric was developed, calibrated and compared to SIRANE results using a set of parameters (local meteorological data, characteristics of industrial sources, e.g. emission intensity and stack height), by calculating weighted kappa statistics (wκ) and coefficient of determination (R^2^). Furthermore, as performance evaluation, the final GIS-based metric was tested to assess atmospheric exposure to cadmium.

**Results:**

The concordance between the GIS-based metric and the dispersion model for dioxin exposure estimate was strong (wκ median = 0.78 (1st quintile = 0.72, 3rd quintile =0.82) and R^2^ median = 0.82 (1st quintile = 0.71, 3rd quintile = 0.87)). We observed similar performance for cadmium.

**Conclusions:**

Our study demonstrated the ability of the GIS-based metric to reliably characterize long-term environmental dioxin and cadmium exposures as well as the pertinence of using dispersion modelling to construct and calibrate the GIS-based metric.

**Electronic supplementary material:**

The online version of this article (10.1186/s12940-019-0446-x) contains supplementary material, which is available to authorized users.

## Background

Outdoor air pollution has been consistently linked to a range of adverse health effects, including cancer and has been estimated responsible for 3.1 million premature annual deaths worldwide [1]. Outdoor air pollution is a mixture of multiple pollutants originating from a large variety of sources, including various carcinogens classified as carcinogenic (Group 1) or probably carcinogenic (Group 2A) to humans by the International Agency for Research on Cancer (IARC) in 2013 [2]. Several recent epidemiological studies investigated the association between outdoor air pollution and breast cancer risk but results remain inconsistent. For traffic-related air pollutants (nitrogen oxides, particulate matters and polycyclic aromatic hydrocarbons), case-control studies highlighted positive associations [3–8] while prospective cohort studies did not report significant associations [6, 9–12]. Only few studies investigated the effect of airborne exposure to dioxins and cadmium (Cd) on breast cancer risk and overall results are inconclusive and need to be further investigated [6, 13, 14]. Finally, inconsistency across results of studies on xenoestrogen exposure in ambient air and breast cancer risk from the literature could be explained by methodological limitations, including lack of historical measurements and insufficient statistical power [15–18]. The multiplicity of exposure sources and the latency between exposure and cancer occurrence represent major challenges and require to precisely characterize the spatial-temporal variability of exposures over large areas and long time-periods. In numerous studies, the lack of past residential history and historical air pollutant exposure assessment at a fine spatial and temporal scales may have resulted in exposure misclassification, hence likely to have contributed to imprecise risk estimates [13, 15, 19]. Also, exposure to dioxins in the general population occurs through emissions in the atmosphere of particles with a large size range leading to exposure through direct inhalation, in particular in earlier years, but also from consumption of contaminated fat-rich food or dermal contact via the wet and dry deposition of particle and the contamination of the food chain [20]. While numerous facilities, including metal industries and cement kilns are likely to emit dioxins [17, 20, 21], the majority of published studies restricted exposure assessment to incinerators [15, 22, 23]. Moreover, information on the evolution of the facility technologies and activity over time is needed to precisely assess long-term dioxin exposure [24].

Furthermore, to overcome the lack of measurement data, previous studies investigating the impact of dioxin exposure on linear distance or presence/absence of the source as a measure of exposure [15, 17, 25, 26]. Yet, the use of these kinds of proxies has been shown to be subject to substantial misclassification [15]. Therefore, dispersion modelling, is considered more reliable to accurately assess exposure with a high spatial resolution [15].

While the spatial coverage of ambient air quality monitoring networks has steadily increased in recent years, different approaches have been developed to adequately represent the spatial variation of pollutants and reconstruct retrospective exposure for earlier periods, including atmospheric dispersion modeling [27], land use regression (LUR) models [28–30] and Geographical Information Systems (GIS). In the case of dioxins, the lack of monitoring data in France as input source and the sharp decrease in emissions over the past 30 years [31] limit the use of LUR modelling to assess dioxin exposure over the French territory. Furthermore, the use of atmospheric dispersion models has to face several difficulties due the fact that the pollutant sources and receptors are distributed over wide areas (the whole France). Performing simulations in domains of these sizes requires significant computational resources. The applicability of these methods (LUR and deterministic models) to assess dioxin and cadmium exposure, for which the number of measurements is extremely limited in time and space and for which there is no comprehensive historical emission inventory. Consequently, the use of GIS opens up a perspective for the characterization of atmospheric exposures to these pollutants in epidemiological studies.

GIS are being increasingly used in environmental epidemiological studies and are based on the residential proximity to distinct types of environmental exposure sources (e.g. industrial facilities and traffic roads) considered as an exposure surrogate. Moreover, GIS allows integrating meteorological and topographical parameters influencing pollutant dispersion, into a GIS-based exposure metric [32–35]. The positional accuracy of subjects’ residences is a key requisite to avoid exposure misclassification [36, 37].

The objective of this study was to develop and calibrate, through a comparison with a Gaussian dispersion model, a GIS-based metric assessing long-term airborne dioxin exposure of participants in a case-control study nested in the French E3N cohort (Etude Epidémiologique auprès de femmes de la Mutuelle Générale de l’Education Nationale) in order to investigate the association with breast cancer risk.

## Methods

### Study population

The E3N study is an ongoing prospective cohort involving 98,995 French female volunteers, born between 1925 and 1950, and members of a national teachers’ health insurance plan, and aimed to identify female cancer risk factors [38]. Since 1990, participants completed self-administered questionnaires, mailed every 2–3 years, on health status, medical history and main cancer risk factors (hormonal, reproductive, dietary and lifestyle-related factors). The E3N cohort is the French component of the European Prospective Investigation into Cancer and nutrition (EPIC) study [39, 40].

For this nested case-control study, 5455 incident invasive breast cancer cases (confirmed by pathology report) and 5455 matched controls were selected from the E3N cohort. Participants were included if they had filled in their home address at baseline, lived in the metropolitan French territory between 1990 and 2008, and had not reported cancer at baseline. According to an incidence density sampling, one control per case was randomly selected and matched to cases for age, department of residence, menopausal status, date of recruitment or blood draw and existence of a biological sample.

### Preliminary steps to the GIS-based metric

The GIS-based assessment of industrial airborne exposure to dioxins was based on a national inventory of dioxin sources and estimation of the annual dioxin emissions, the geocoding of dioxin sources and of the residential history of study participants over the study period.

#### Dioxin industrial sources: Inventory and characterization

A detailed retrospective inventory of industrial sources likely to emit or to have emitted dioxins between 1990 and 2008 over the whole France was carried out, taking into account waste incineration, medical waste incineration, metal production, heat and power generation, production of mineral products, chemicals and consumer goods, and crematoria. Dioxin sources were identified through institutional and national databases, namely GEREP (annual emission reporting on pollutant and greenhouse gas releases), IREP (French National Registry of Pollutant Emissions) and ICPE (Inventory of French classified facilities). Industrial unions, nationally recognized associations and whistleblowers were contacted to identify additional facilities, in particular for earlier years and a structured questionnaire was sent to these identify facilities in order to collect additional information on technical characteristics. From 1990 to 2008, a total of 2626 dioxin sources were inventoried over the French territory.

Dioxin releases mostly depend on the combustion process and conditions, the type of material burned and the flue gas treatment. Along with the inventory, technical characteristics were collected, including geographic location of the facilities (geographic coordinates and addresses), operation periods and rates, stack height, process characteristics and flue gas cleaning technologies for the different periods. Using the technical characteristics of dioxin sources, intensity of dioxin emissions were estimated using the Standardized Toolkit for Identification and Quantification of Dioxin and Furan Releases developed by the United Nation Environmental Program [41]. The Toolkit allowed for a classification of dioxin emissions according to activity sectors and operating characteristics. The industrial sources inventoried were classified into Toolkit categories and a dioxin emission factor (in g-TEQ/t) was assigned. For each distinct operation periods, annual dioxin emission intensity (in g-TEQ/year) was estimated by multiplying the emission factor by the operation rate. A general decrease was observed in the dioxin source annual average emissions (Table 1) due to the improvement of gas cleaning technologies.

**Table 1:** Average dioxin (g-TEQ/year) and cadmium (kg/year) release estimates of the sources inventoried for 1996, 2002 and 2008 in France, Lyon, Le Havre and Le Bugey

Areas	1996		2002		2008	
	*n*	mean ± SD	*n*	mean ± SD	*n*	mean ± SD
Dioxins						
France	918	2.76 ± 8.08	850	0.66 ± 2.64	854	0.08 ± 0.42
Lyon	23	3.08 ± 12.68	24	0.58 ± 1.91	28	0.02 ± 0.03
Le Havre	7	7.07 ± 11.93	8	4.13 ± 11.42	7	0.05 ± 0.05
Le Bugey^a^	4	1.63 ± 2.27	4	0.51 ± 0.38	4	0.31 ± 0.22
Cadmium						
France	963	24.4 ± 121.1	906	20.7 ± 121.2	909	9.5 ± 51.5
Lyon	23	11.6 ± 31.5	24	6.1 ± 5.7	28	1.1 ± 2.4
Le Havre	7	25.3 ± 25.6	8	11.1 ± 11.5	7	4.7 ± 4.9
Le Bugey^a^	4	27.8 ± 41.9	4	15.0 ± 23.4	4	3.5 ± 4.4

*n*: number of industrial sources; ^a^For the need of the calibration three of the four sources in Le Bugey were virtual; SD: Standard deviation

#### Geocoding of the residential history and sources

As this current work is a part of a GIS-based metric assessing long-term airborne dioxin exposure of the participants of the whole cohort-nested case-control study, location of facilities and geocoding of the participants’ residential history was performed for the whole France, using ArcGIS software (ArcGIS Locator, ESRI, Redlands, CA, USA), BD Adresse for ArcGIS and its reference street network database, BD Adresse® (National Geographic Institute, IGN, Saint Mandé, France) that includes 26 million addresses. The choice of the geocoding method was based on the accuracy of positioning to limit exposure misclassification and has been described in detail elsewhere [36]. Overall 28,511 residential addresses in metropolitan France, collected from the questionnaires sent between 1990 and 2005, were geocoded; 78.1% of the subject’s residences were geocoded to the address, 26% required manual checking and among them 17.4% were corrected.

Facilities identified through the inventory were located based on their geographic coordinates when available, or geocoded using the addresses collected. All automatically geocoded locations were manually checked and repositioned at the stack using current and historical aerial photography from IGN. Facilities for which the stack was not visible on current or past aerial images, were located as accurately as possible in descending order according to positional accuracy, *i.e.* at the centroid of the building; at the centroid of the parcel; or at the town hall of the municipality. Among the 2626 sources inventoried in France between 1990 and 2008, 82% were positioned at the stack, 13% at the centroid of the building and 5% at the parcel.

### Development of the GIS-based metric

#### Identification of relevant parameters

Based on the review of relevant publications in the literature [17, 32, 34, 35, 42–44] and previous work on dioxin and cadmium modelling [31], the following parameters were included in the GIS-based metric to characterize exposure and classify study subjects according to their airborne dioxin exposure from industrial sources (Table 2): subject’s residence-to-source distance, wind direction and speed, exhaust smoke velocity and stack height. For all parameters, a setting sample was tested to identify the relevant combination of parameters (Table 2). The selected parameters were combined with the sources and subject locations, the sources’ annual emission intensity (in g-TEQ/year) and the exposure duration (in years).

**Table 2:** Parameters and modalities tested to calibrate the GIS-based metric

Type	Parameters	Modalities tested
Geographical	Impact zone of dioxin industrial emissions	Circular buffer with 3, 5 and 10 km radius
	Residence-to-source distance decline	$\frac{1}{d};\frac{1}{d^{1.5}};\frac{1}{d^2};\frac{1}{d^3};{e}^d;\frac{e^d}{d};{e}^{-d}.d$
Meteorological	Wind frequency	Equal wind rose segment sizes (CADDs) of 90°, 30°, and 10°± Weighted contribution of adjacent segments
	Wind speed	Variation of the residence to source distance decline pattern according to the hourly wind speed
Technical	Exhaust smoke velocity	$\frac{1}{v}$ *;* $\frac{1}{v^2}$
	Stack height	$\frac{1}{h};\frac{1}{h^{0.5}};\frac{1}{h^2}$

*d: residence to source distance; v: exhaust smoke velocity; h: facility’s stack height*

#### Integration of the selected parameters

Proximity to dioxin sources is a key parameter to assess individual dioxin exposure [15]. Based on the literature, three different buffer sizes were tested in the calibration step, corresponding to a circular buffer around each dioxin source of respectively 3 km, 5 km and 10 km [17, 42–44]. A matrix of residence-to-source distance was calculated using the Point-to-Point function in ArcGIS software. Subjects residing outside the buffer were considered as non-exposed [44]. Inside the buffer, the decrease in dioxin concentrations was calculated testing different residence-to-source distance decline patterns (Table 2).

Pollutant atmospheric dispersion depends on meteorological conditions, in particular wind direction and speed. We included wind parameters in the GIS-based metric using data from the French national meteorological service, METEO France, based on 727 areas of homogeneous weather pattern (AHWP) in metropolitan France. To take into account continuity of meteorological monitoring over the study period (1990–2008), the 727 AHWP were grouped, in collaboration with METEO France, into 223 meteorological areas according to the proximity to the reference station, homogeneity of weather pattern and type of area (plain, mountain, hillside and valley). The measurement stations provided information on wind direction and speed between 1990 and 2008 on an hourly basis with an average completeness of data around 90% for the whole study period. Each inventoried industrial source was assigned to a reference meteorological station representative of the local weather conditions. For a given year, if the completeness was below 75%, data from the nearest meteorological station were used.

**Fig. 1:**
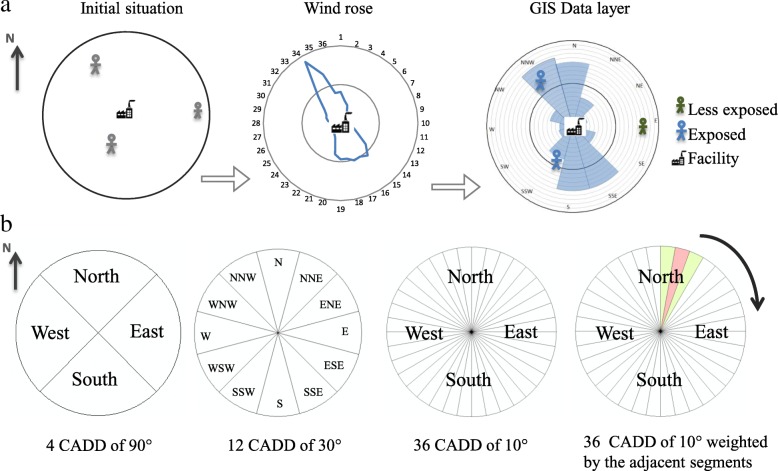
**a** From a wind rose to the creation of contributed area for dioxin dispersion (CADD). **b** Degrees of CADD tested to consider wind directions in the GIS metric

To integrate wind directions into the GIS, a GIS data layer named contributing area for dioxin dispersion (CADD), was created for each dioxin source, based on equal segments of the wind rose and proportion of annual wind blow and speed at each segment (Fig. 1). In the calibration step, we tested CADDs of 10°, 30° and 90°. Furthermore, for CADDs of 10°, weighted contribution (50% and 25%) of adjacent segments was assessed [44]. To take into account the wind speed, we reported the average annual wind speed for each segment and adjusted the decrease in dioxin concentration according to this average wind speed. For a given subject, exposure was estimated based on the CADD in which the subject’s residence was located. The process was managed with ArcMap 10.1.

### GIS-based metric calibration

#### Specific areas and periods selected for metric calibration

We restricted the calibration of the GIS-based metric to three French geographical areas presenting typical topographical and meteorological patterns and distinct numbers and types of dioxin sources (Fig. 3), representative of the living environment of the majority of the E3N subjects: Lyon (a non-mountainous highly urban area), Le Bugey (a rural area) and Le Havre (a costal medium-size urban area). We further selected three distinct years over the study period with a 6-years gap (1996, 2002 and 2008) presenting, for each area, different emission intensities and meteorological parameters.

In the Lyon area, addresses were collected from the E3N questionnaires sent in 1997, 2002 and 2005 (respectively for 1996, 2002 and 2008 scenarios). Due to residential mobility, loss to follow-up and, exclusion of case-control pairs following breast cancer diagnosis, the number of subjects decreased over the study period (312 subjects in 1996, 173 in 2002 and 68 in 2008). In this area, 80.8% of the study subjects were located at the address level and 16.9% at the street address. The number of E3N subjects residing in Le Bugey and Le Havre was lower (*n* < 30). In order to obtain a relevant number of subjects residences for the present calibration study, 150 simulated subjects’ residences were randomly located in each of these two areas. The accuracy of the participant residential addresses geocoded was similar with the one observed in the whole France (see 2.2.).

Overall, 40 dioxin sources were identified and located in Lyon, 9 in Le Havre and 1 in Le Bugey. All the 50 sources were located at the stack. In Lyon and Le Havre, the number of sources inventoried was sufficient to perform a comparison between the Gaussian model and the GIS-based metric (Table 1). As only a single industrial source was inventoried for the Bugey, we added three virtual sources with differing parameters (stack height, smoke exit velocity, emission intensity, etc.) and annually varying dioxin emissions. The choice of technical parameters and emissions of these three virtual sources were based on average values observed during the inventory step, for three emission domains: heat and power generation, metal production and crematoria.

Annual average dioxin emission estimates for each area and year are described in Table 1. Similarly to that at the national level, a decrease is observed, from 1996 to 2008, for each of the selected areas. These changes in emissions were due to new emission policies, applied at the end of the 90’s [24]. The observed decrease is sharper for Le Havre (from 4.13 g/year in 2002 to 0.05 g/year in 2008) than for the two other sites, and is mainly due to the closing of a major industrial source after 2002.

#### Modelling of airborne dioxin exposure

Modelling of dioxin atmospheric dispersion was performed in Lyon, Le Bugey and Le Havre, for the three distinct periods (1996, 2002, and 2008) using the SIRANE atmospheric dispersion model. SIRANE is an urban dispersion model that integrates a specific module to simulate pollutant dispersion within a built environment [45, 46], considering local meteorological conditions and geometry of the streets. The SIRANE model has been validated by means of wind tunnel experiments [47, 48] and open field measurement data [45, 49]. Note that a detailed validation of the model, based on NO2 concentration levels, was performed over the whole Lyon urban agglomeration for the year 2008, *i.e.* one of the three domains considered in this study. Details on SIRANE dioxin modelling results are available in Additional file 1.

Average annual dioxin concentrations (in fg-TEQ/m^3^) were calculated at each E3N subject’s residence location for each year and categorized into quintiles according to the average dioxin concentration. Modelled dioxin concentrations served as reference for the calibration and validation of the GIS-based metric. Modelled dioxin concentrations for 2008 in Lyon, were compared to weekly average concentrations provided by a monitoring station located in the city center of Lyon since 2007 [31].

### Performance evaluation

Once selected the parameters to be integrated in the GIS-based metric and best-performing parameter combination, two evaluations were completed to assess the performance of the GIS-based metric.

For the first performance evaluation, 150 new virtual subjects were randomly distributed in each of the three areas. The periods (1996, 2002 and 2008), source locations and emissions remained unchanged. Given the size of the Lyon area (34 km × 30 km), and the variation of population density over the area, the distribution was weighted according to population density in the Lyon area.

The second performance evaluation was achieved to assess the performance of the GIS-based metric to assess exposure to pollutants other than dioxins. For this purpose, we applied the GIS-based metric to assess cadmium exposure. Cadmium industrial sources were inventoried through institutional and public databases, industrial unions and nationally recognized associations. Using emission factors provided by the OMINEA database (Organization and Methods of the National Inventories of the Atmospheric Releases in France), from the Inter-professional Technical Centre for studies of Air Pollution (CITEPA), and technical parameters collected through similar steps as for dioxins, annual cadmium emissions were estimated for each industrial source. A total of 2686 cadmium emitting sources were inventoried over the French national territory from 1990 to 2008. At the national level, annual cadmium average emissions decreased from 24.4 kg/year to 9.5 kg/year between 1996 and 2008 (Table 1). More details are provided in a previous publication [31]. In the three selected areas, cadmium emission estimates showed similar levels and trends to those observed at the national level (Table 1).

As for dioxins, the GIS-based metric was applied in Lyon, Le Bugey and Le Havre for 1996, 2002 and 2008 and modelling of cadmium atmospheric dispersion was performed using SIRANE for the same years and areas. Details on SIRANE cadmium modelling results are available in Additional file 2.

### Statistical analyses

The GIS-based metric was defined through comparison with annual dioxin concentrations estimated by SIRANE, for each scenario, which allowed selecting the most relevant parameters and their combination to be included in the GIS-based metric.

For the development of the GIS-based metric and its performance evaluation, we compared the categorical dioxin exposure classification (based on quintiles) of study subjects between the GIS-based metric and the SIRANE dispersion modelling for the three locations (Lyon, Le Bugey, Le Havre) and the three different years (1996, 2002 and 2008),

Agreement between quintiles of dioxin concentrations from modelling and quintiles of the GIS-based metric estimates was calculated using weighted kappa coefficients (wκ) and their 95% confidence intervals (95% CI). Weighted kappa coefficients assign less importance to discrepancies between adjacent quintiles and higher weight to larger discrepancies [15]. The determination coefficient R^2^ was also computed for each scenario. Analyses were performed using SAS software version 9.4 (SAS Institute Inc., Cary,NC).

## Results

### Calibration of the GIS-based metric

We observed higher wκ for a buffer size of 10 km (wκ ranging from 0.42 to 0.71 depending on the parameter combinations) compared to buffers of 3 km and 5 km (wκ ranging from 0.31 to 0.42 and from 0.34 to 0.60 for 3 km and 5 km, respectively). Taking into account wind direction, using CADDs, increased the metric performance, in particular for CADDs with 10° wind rose segments (Table 3 for Lyon area; see Additional file 3 for Le Havre area). The highest agreement was obtained for an inverse subject’s residence-to-source square distance weighting. The addition of wind speed parameters decreased the agreement: wκ (95% CI) ranging from 0.71 (0.67, 0.76) to 0.81 (0.79, 0.88) without wind speed and from 0.58 (0.49, 0.66) to 0.71 (0.70, 0.82) after integration of wind speed in the Lyon scenario (see Additional file 4).

**Table 3:** Weighted kappa coefficients (wκ) and corresponding 95% confidence intervals (95% CI) for the calibration in the Lyon area (1996, 2002 and 2008)

Integration of wind direction into the GIS-based metric ((wκ (95% CI))							
Distance decline	Year	Without integration of wind direction	90°; 4 CADD	30°; 12 CADD	10°; 36 CADD	10° with adjacent segments; weighted at 25%	10° with adjacent segments; weighted at 50%
1/d	1996	0.64 (0.59, 0.69)	0.65 (0.59, 0.70)	0.57 (0.51, 0.63)	0.64 (0.59, 0.69)	0.56 (0.50, 0.63)	0.56 (0.50, 0.62)
	2002	0.61 (0.54, 0.68)	0.63 (0.55, 0.70)	0.61 (0.54, 0.68)	0.60 (0.52, 0.67)	0.60 (0.52, 0.67)	0.60 (0.53, 0.68)
	2008	0.71 (0.61, 0.82)	0.48 (0.34, 0.63)	0.56 (0.42, 0.70)	0.69 (0.58, 0.80)	0.67 (0.56, 0.78)	0.69 (0.59, 0.80)
1/d^2^	1996	0.61 (0.55, 0.66)	0.70 (0.66, 0.75)	0.76 (0.72, 0.81)	0.75 (0.71, 0.79)	0.71 (0.67, 0.76)	0.71 (0.67, 0.76)
	2002	0.60 (0.53, 0.68)	0.71 (0.65, 0.78)	0.80 (0.75, 0.85)	0.83 (0.76, 0.88)	0.82 (0.78, 0.87)	0.84 (0.79, 0.88)
	2008	0.64 (0.52, 0.75)	0.62 (0.49, 0.74)	0.66 (0.54, 0.77)	0.83 (0.75, 0.90)	0.79 (0.70, 0.88)	0.81 (0.72, 0.89)
e^-d^	1996	0.52 (0.46, 0.59)	0.63 (0.56, 0.67)	0.63 (0.57, 0.68)	0.39 (0.32, 0.46)	0.65 (0.58, 0.70)	0.65 (0.60, 0.70)
	2002	0.52 (0.43, 0.60)	0.65 (0.57, 0.72)	0.68 (0.60, 0.75)	0.32 (0.23, 0.42)	0.65 (0.58, 0.73)	0.66 (0.59, 0.74)
	2008	0.58 (0.45, 0.71)	0.60 (0.47, 0.73)	0.62 (0.49, 0.74)	0.43 (0.27, 0.58)	0.71 (0.61, 0.81)	0.71 (0.61, 0.81)
e^-d^/d	1996	0.48 (0.41, 0.55)	0.57 (0.51, 0.63)	0.59 (0.53, 0.65)	0.64 (0.59, 0.69)	0.59 (0.54, 0.65)	0.59 (0.54, 0.65)
	2002	0.52 (0.42, 0.61)	0.63 (0.54, 0.71)	0.65 (0.57, 0.73)	0.60 (0.52, 0.67)	0.65 (0.57, 0.73)	0.64 (0.56, 0.72)
	2008	0.58 (0.46, 0.70)	0.54 (0.40, 0.68)	0.60 (0.47, 0.73)	0.69 (0.58, 0.80)	0.62 (0.49, 0.74)	0.62 (0.50, 0.74)
1/d^1,5^	1996	0.63 (0.57, 0.68)	0.71 (0.66, 0.75)	0.70 (0.65, 0.75)	0.72 (0.68, 0.76)	0.66 (0.61, 0.70)	0.66 (0.61, 0.71)
	2002	0.61 (0.54, 0.68)	0.73 (0.67, 0.79)	0.74 (0.68, 0.80)	0.75 (0.69, 0.81)	0.73 (0.67, 0.79)	0.73 (0.67, 0.79)
	2008	0.66 (0.54, 0.77)	0.58 (0.46, 0.70)	0.62 (0.49, 0.75)	0.85 (0.78, 0.92)	0.79 (0.70, 0.88)	0.79 (0.70, 0.88)
1/d^3^	1996	0.52 (0.46, 0.58)	0.64 (0.58, 0.69)	0.67 (0.62, 0.72)	0.69 (0.65, 0.74)	0.67 (0.62, 0.72)	0.68 (0.63, 0.73)
	2002	0.55 (0.47, 0.64)	0.67 (0.60, 0.74)	0.77 (0.70, 0.83)	0.77 (0.70, 0.83)	0.75 (0.69, 0.81)	0.74 (0.68, 0.81)
	2008	0.58 (0.45, 0.71)	0.60 (0.46, 0.74)	0.62 (0.48, 0.75)	0.66 (0.54, 0.77)	0.69 (0.58, 0.81)	0.69 (0.58, 0.81)
e^-d^*d	1996	0.55 (0.49, 0.61)	0.64 (0.59, 0.69)	0.67 (0.63, 0.72)	0.24 (0.16, 0.32)	0.69 (0.65, 0.74)	0.69 (0.64, 0.73)
	2002	0.55 (0.47, 0.63)	0.68 (0.62, 0.75)	0.74 (0.68, 0.81)	0.16 (0.06, 0.27)	0.74 (0.68, 0.80)	0.74 (0.68, 0.80)
	2008	0.60 (0.47, 0.72)	0.62 (0.50, 0.74)	0.69 (0.58, 0.81)	0.31 (0.15, 0.47)	0.69 (0.60, 0.79)	0.69 (0.59, 0.79)

*d* residence to source distance, *CADD* contributing area for dioxin dispersion

The integration of the source technical parameters (exhaust smoke velocity and stack height) did not further improve agreement between the categorical dioxin exposure classification by the two methods (see Additional files 5 and 6), except for Le Havre in 2008 where the integration of the stack height of the major industrial source (240 m) led to considerable improvement in the agreement between the GIS-based metric and the dispersion modelling (wκ (95% CI) from 0.64 (0.59, 0.71) and 0.78 (0.72, 0.84) without and with integration of stack height respectively). Given the absence of impact of the stack height on the weighted kappa coefficients in all other scenarios and the low completeness of data for stack height at the national level (36%), it was decided to integrate into the GIS-based metric only stack heights above 90 m, corresponding to 3 times the median stack height of the 2626 sources over the whole France. Data were available for all sources with stack height above 90 m.

**Fig. 2:**
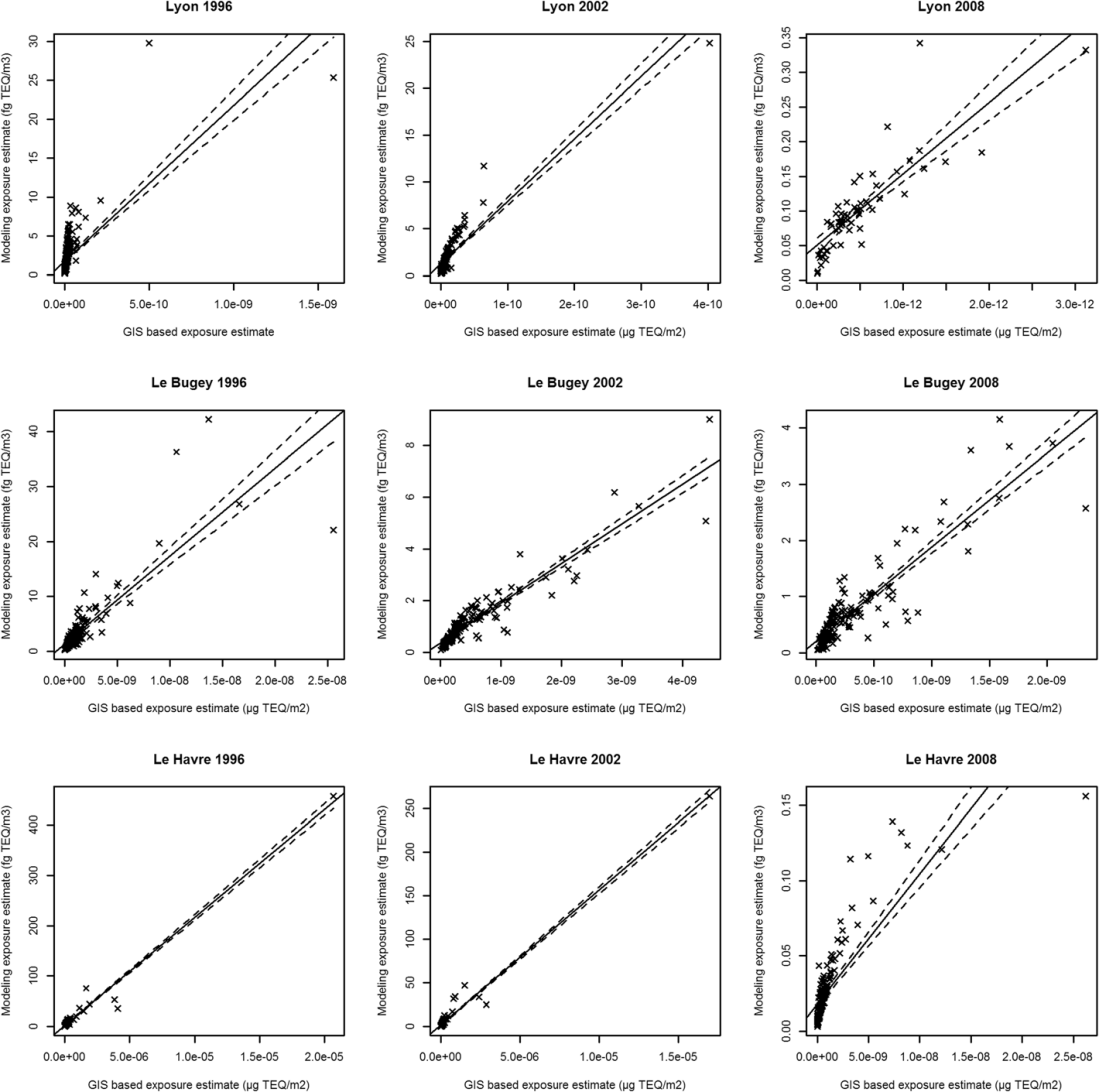
Scatterplots of subject classification using the GISbased metric and the SIRANE model

**Fig. 3:**
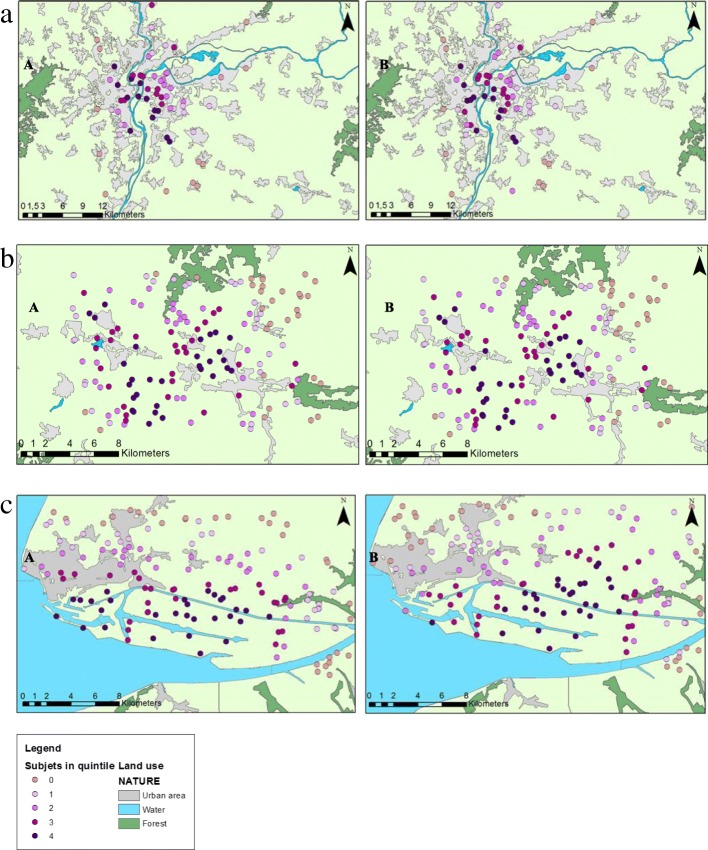
**a** Lyon area, subject classification into quintiles of exposure, calibration set, 2008. **b** Le Bugey area, subject classification into quintiles of exposure, calibration set, 2008. **c** Le Havre area, subject classification into quintiles of exposure, calibration set, 2008

Based on the performance of the nearly 80 different parameter combinations in the nine calibration scenarios (3 areas over 3 years, see Figs. 2 and 3), we retained the following formula for the GIS-based metric:

$$ {\displaystyle \begin{array}{l}\mathrm{GIS}-\mathrm{based}\kern0.5em \mathrm{metric}\kern0.5em \left(\mathrm{g}-\mathrm{TEQ}.{\mathrm{m}}^{-2}\right)=\kern0.5em \sum \limits_{\mathrm{j}}^{\mathrm{J}}\ \sum \limits_{\mathrm{i}}^{\mathrm{I}}\kern0.5em {\mathrm{t}}_{\mathrm{j}}\times \kern0.5em \frac{1}{{\mathrm{d}}_{\mathrm{i}\mathrm{j}}^2}\kern0.5em \times \kern0.5em \mathrm{E}{\mathrm{I}}_{\mathrm{i}}\kern0.5em \times {\mathrm{F}}_{\mathrm{i}}\kern0.5em \times \kern0.5em {\left(\frac{{\mathrm{h}}_{\mathrm{m}\mathrm{edian}}}{{\mathrm{h}}_{\mathrm{i}}}\right)}^{\mathrm{a}}\kern0.5em \left(\mathrm{i}\right)\\ {}\kern38em \end{array}} $$

^a^ if h_i_ is greater than 90 m

where j is the place of residence (j = 1,…,J), i is the industrial source (*i* = 1,…,I), EI_i_ is the annual dioxin emission intensity (in g-TEQ/year), t_j_ is the exposure duration (in year), d_ij_ is the residence-to-source distance (in m), F_i_ is the percentage of time with the wind on the CADD of the subject location, h_i_ is the stack height (in m) and h_median_ is the median value of the other sources’ stack height (in m) in a 10 km buffer.

Using the formula (*i*), the nine calibration scenarios, yielded wκ (95% CI) ranging from 0.71 (0.67, 0.76) to 0.84 (0.79, 0.88), corresponding to a “substantial” to “almost perfect” agreement between the categorical dioxin exposure classification into quintiles by the GIS-based metric, and the SIRANE dispersion modelling (Fig. 3). The R^2^ ranged from 0.68 to 0.90 for the same scenarios (Table 4). The scatterplots (Fig. 2) illustrates the ability of the GIS-based metric to provide robust estimates of the subject’s exposure in comparison to modelling results.

**Table 4:** Weighted kappa coefficients (wκ), corresponding 95% confidence intervals (95% CI) and R^2^ obtained with the final metric for Lyon, Le Havre and Le Bugey for 1996, 2002 and 2008

Years	Areas	Lyon	Le Bugey	Le Havre
Calibration				
1996	wκ (95% CI)	0.71 (0.67, 0.76)	0.79 (0.73, 0.85)	0.74 (0.68, 0.80)
	R^2^	0.78	0.83	0.68
2002	wκ (95% CI)	0.84 (0.79, 0.88)	0.82 (0.76, 0.87)	0.77 (0.72, 0.83)
	R^2^	0.83	0.90	0.69
2008	wκ (95% CI)	0.81 (0.72, 0.89)	0.73 (0.66, 0.79)	0.78 (0.72, 0.84)
	R^2^	0.87	0.77	0.84
Performance evaluation 1: new virtual subjects randomly distributed^a^				
1996	wκ (95% CI)	0.71 (0.64, 0.78)	0.86 (0.81, 0.90)	0.71 (0.64, 0.78)
	R^2^	0.95	0.86	0.30
2002	wκ (95% CI)	0.67 (0.60, 0.74)	0.86 (0.81, 0.91)	0.74 (0.68, 0.80)
	R^2^	0.94	0.55	0.80
2008	wκ (95% CI)	0.58 (0.49, 0.66)	0.84 (0.79, 0.89)	0.79 (0.73, 0.84)
	R^2^	0.88	0.75	0.62
Performance evaluation 2: cadmium emissions				
1996	wκ (95% CI)	0.69 (0.64, 0.73)	0.83 (0.78, 0.87)	0.72 (0.67, 0.78)
	R^2^	0.74	0.71	0.65
2002	wκ (95% CI)	0.86 (0.82, 0.91)	0.82 (0.77, 0.87)	0.77 (0.71, 0.82)
	R^2^	0.83	0.86	0.66
2008	wκ (95% CI)	0.73 (0.64, 0.83)	0.69 (0.63, 0.76)	0.74 (0.68, 0.80)
	R^2^	0.82	0.83	0.80

^a^Adjusted on population density for Lyon's scenario

### Performance evaluation of the GIS-based metric

Once established, the final GIS-based metric (*i*) was applied to the new samples of virtual subjects randomly located in Lyon, Le Bugey and Le Havre for 1996, 2002 and 2008. The calibration scenarios yielded wκ (95% CI) ranging from 0.58 (0.49, 0.66) to 0.86 (0.81, 0.91) (Table 4). Weighted kappa were below 0.6 (0.58) for one scenario (Lyon, 2008). The determination coefficients ranged from 0.30 to 0.94 for these nine scenarios (Table 4) with one scenario under 0.5 (Havre, 1996; R^2^: 0.3) and 4 scenarios above 0.85.

The GIS-based metric (*i*) was further applied to estimate airborne cadmium exposure (Table 4). We observed a “substantial” to “almost perfect” agreement for categorical dioxin exposure classification (quintiles) between the GIS-based metric and the SIRANE modelling with wκ (95% CI) ranging from 0.69 (0.64, 0.73) to 0.86 (0.82, 0.91). The wκ remained consistent across sites, periods, emission intensities and number of sources. The R^2^ for these nine scenarios ranged from 0.66 to 0.86.

## Discussion

GIS are being increasingly used in epidemiological studies to compute exposure surrogates based on distance between study population and exposure sources or using more advanced methods integrating meteorological and topographical data, residential history as well as characteristics of industrial sources [17, 34, 42–44]. We developed a GIS-based metric in this way, filling methodological gaps of the existing literature to improve accuracy of airborne dioxin exposure estimates. To our knowledge, this is the first study calibrating a GIS-based metric evaluating its performance to estimate dioxin or more largely, air pollutant exposure, from industrial sources, to be used in an epidemiological study, through comparison to the SIRANE model [49].

The combination of parameters demonstrated consistently reliable estimates for the two performance evaluations with differing number of sources, subjects, meteorological and topographical conditions. Weighted kappa coefficients indicated “substantial” to “almost perfect” [50] agreement with the modelled estimates, except for one scenario (Lyon, 2008; first performance evaluation set). The relative poor performance in this scenario (wκ (95% CI): 0.58 (0.49, 0.66)) may be explained by the situation for Lyon in 2008 involving a high density of sources (*n* = 33) with very low and homogenous source emissions increasing the difficulty to differentially classify study subjects into quintiles [51]. Note that in this scenario, we obtained a high determination coefficient (R^2^: 0.88).

Likewise, the values of the coefficient of determination (R^2^) demonstrated reliable estimates by the GIS-based metric with median values of 0.80. Only one scenario (Le Havre, 1996, first performance evaluation set) showed a poor performance. This observation can be explained by two outliers (out of the 150 virtual subjects for this area) that were 10 times more exposed than all other subjects and underestimated by the GIS-based metric. The value of R^2^ increased from 0.30 to 0.95 after the exclusion of these two subjects, which were randomly located. It is worth noting that for this same situation, a substantial agreement was obtained for the categorical exposure classification (wκ (95% CI): 0.71(0.64, 0.78)). Regarding the large range of R^2^ obtained, it should be noted that the determination coefficient can be highly influenced by a few subjects with extreme values.

Pronk et al. studied dioxin exposure using a GIS but did not perform calibration or validation [17]. The performance observed in our study for parameter combinations such as used by Pronk et al. 2013 (inverse distances squared-weighted emission, winds not taken into account and buffer limiter to 5 km), was much lower, with wκ ranging from 0.31 to 0.45.

Overall, the level of performance of our metric is comparable to other studies conducted in Europe on more frequently studied pollutants [15, 32, 34, 42]. Unlike dioxins and cadmium, numerous measurement are available for NO_2_ [42], PM_10_ [15, 32] and black smoke [34], and facilitates calibration and validation. Cordioli et al. (2013), using PM_10_ as a tracer for incinerator pollutant emissions, evaluated the agreement of categorical exposure classifications of subjects between PM_10_ concentration maps and different exposure methods, including a simple indicator based on distance between exact address location and incinerator. Using this simple indicator, the authors obtained a wκ of 0.61. We observed similar results when our GIS-based metric was only based on source-subject distance (wκ (95% CI) ranging from 0.61 (0. 59, 0.96) to 0.78 (0.73, 0.84)). Three studies conducted a calibration using measurements [32, 34, 42] and two of them completed a performance evaluation. Gulliver and Briggs (2011) obtained a good agreement between ambient air measurements and metric estimates (R^2^: 0.67–0.77) for annual PM10 concentrations in London [32]. These results were comparable to performance realised by a Gaussian model (R^2^: 0.71–0.77). Vienneau et al. (2009), yielded a determination coefficient of 0.60, using a GIS-based moving window approach, in comparison with NO_2_ measurements across Europe but provided an estimation with limited accuracy (1x1 km^2^) [42].

The estimates observed for cadmium demonstrated the ability of the GIS-based metric to assess, as for dioxins, pollutant exposures from industrial sources with behaviours similar to dioxins, *i.e.* pollutants with particle size around 1 to 10 μm and absence of chemical reaction in the atmosphere. Note that, the SIRANE model for both pollutants used a similar setting (pollutant modelled as a passive scalar with an average diameter of 1 μm) but with different average densities (dioxins: 321.9 g/mol; cadmium: 112.4 g/mol) [31].

Strengths of our study included the use of a GIS and its application on a large area [34, 42], over a long and retrospective time-period, at the individual subject’s address and considering the residential history over the study period [13, 15]. Moreover, they suggest that GIS-based metrics provide a robust alternative to LUR models in case studies with few measured data (limiting the use of LUR models) or wide domains with large number of sources (requiring high computational resources for the use of atmospheric dispersion models). These results show that besides the application for epidemiological purpose, this tool can be use in numerous contexts especially in environmental impact assessment studies where it will be less complex and faster to apply than deterministic or statistical models.

Our GIS-based metric required a retrospective inventory of industrial sources, the estimation of their emission intensity, the geocoding of the participants’ residential history and of the industrial sources, and the computation of local meteorological data and source technical parameters in the GIS. The highest agreement of the parameters combination with the dispersion model was reported for the inversed square residence-to-source distance, as observed in three other studies [17, 34, 42]. The buffer size around sources was set at 10 km, which is consistent with the literature, with buffer sizes ranging from 3 to 10 km for industrial sources [17, 42, 44]. As the exposure due to traffic was not included in the GIS-based metric, smaller buffer sizes were not retained for the current study. Furthermore, while the parameters integrated in our GIS-based metric are consistent with several studies from the literature [15, 32, 35, 43], the inclusion of the wind speed in the parameters’ combination of the GIS-based metric, did not further improve the agreement statistics despites wind speed being known to impact pollutant dispersion, and this may constitute a possible source of error. While other studies integrated wind direction [44] or both wind direction and wind speed in their metric [32, 42, 43], no other study evaluated the impact of wind speed on the metric performance. Similarly, we did not identify studies that evaluated the impact of stack height or other industrial sources technical parameters on the metric performance. Integration of additional parameters, such as pluviometry or outdoor temperature, as well as regional background concentrations [34], may further improve the performance of the GIS-based metric by taking account wet and dry dioxin deposition.

Our study was based on a multi-source approach, considering multiple emission sectors (waste incineration, metal production, cement industries, etc.) and the evolution over time of the facilities’ technical characteristics. In the absence of dioxin monitoring data, emission intensity of the industrial sources was estimated using a standardized tool (http://toolkit.pops.int/). Pronk et al. also used a historical dioxin emission inventory (1987–2000) and a multi-source approach, limited however to few activity sectors [17]. The accuracy of our emission estimates were directly linked to the quality of the information collected from industrial facilities on technical characteristics of the sources. Previous studies often used emission inventories conducted for other purposes [17, 42].

The accuracy of address location may have important implications on misclassification of individual exposure, depending on the spatial concentration gradient of the exposure. Although the residential addresses of the study subjects were not recorded initially to be geocoded and used for the assessment of environmental exposure, their accuracy can be considered precise enough to limit misclassification bias, in particular for urban subjects in the present study [15, 33, 36].

In the case of dioxins, domestic activities are known to poorly contribute to airborne dioxin exposure compared to industrial sources for earlier years [52]. Some non-industrial sources have however become non negligible for more recent periods and may lead to the underestimation of the exposure and to a non-differential misclassification bias. Other punctual and non-industrial sources can emit relatively high amounts of dioxins and cadmium [31, 53], locally and in a short time scale, such as biomass fires, manufactured good burnings, cable burning, outdoor burning and illegal landfills in the early 1990s. These sources could not be considered in this GIS-based metric due to the difficulty of their retrospective inventory, their geolocalization and the estimation of their dioxin emissions. To reconstruct the subjects’ historical dioxin exposure, in an epidemiological context, it seems essential to considered these others types of emissions and the others routes of exposure such as diet due to dioxin wet and dry disposition [54].

The GIS-based metric contributes to improve exposure assessment methodologies. The possibility of taking into account chronic exposures is relevant for the study of a large number of biological pathologies and mechanisms [55]. Moreover, several recent studies have shown the need to study exposures accurately over short periods and specific exposure windows [56]. Ren et al. (2017). have recently shown for PM (whose behaviour in the atmosphere is similar to dioxins in particulate form) that exposure, one month before and after pregnancy increases the risk of birth defects [57]. In addition to long-term exposure assessment, this GIS indicator, thanks to the finesse of the meteorological information collected, is able to estimate exposure at a daily temporal scale over the entire French territory between 1990 and 2008 and thus minimizes potential classification bias of future epidemiological studies.

## Conclusion

In this study, a GIS-based metric has been developed and evaluated in order to estimate the retrospective airborne dioxin exposure of participants of a cohort-nested case-control study. The final metric combined residential distance to facilities, wind direction and proportion of the year blown and technical parameters of the facilities. This combination of parameters showed reliable estimates in comparison to an atmospheric dispersion model [49] across different scenarios. The GIS-based metric also provided reliable estimates for cadmium exposure from industrial sources and might be able to assess exposure to other air pollutants with similar properties and behaviour than dioxins and cadmium (*i.e.* heavy metals, PM_10_ etc.), in particular when monitoring data are lacking. In addition to its use in epidemiology studies, the GIS-based metric may provide a useful tool for environmental impact assessment.

## Additional files

Additional file 1: Boxplot of the average dioxin concentrations (fg-TEQ/m3), modeled at the E3N location in Lyon, Le Havre and Le Bugey for 1996, 2002 and 2008 with the SIRANE model. This figure shows the reparation of subjects’ exposure to dioxin, obtained with the SIRANE model, for 3 years (1996, 2002 and 2008) for the 3 areas (Le Havre, le Bugey, Lyon). (DOCX 582 kb)

Additional file 2: Boxplot of the average cadmium concentrations (ng/m3), modeled at the E3N location in Lyon, Le Havre and Le Bugey for 1996, 2002 and 2008 with the SIRANE model. This figure shows the reparation of subjects’ exposure to cadmium, obtained with the SIRANE model, for 3 years (1996, 2002 and 2008) for the 3 areas (Le Havre, le Bugey, Lyon). (DOCX 737 kb)

Additional file 3: Weighted kappa coefficients and CI95% with different CADD and distance decline patterns in the Le Bugey scenarios. This table shows the variation of the concordance between the two classifications according to the combination of the setting of different parameters (winds direction and distance decline) in Le Bugey scenario. (DOCX 14 kb)

Additional file 4: Weighted kappa coefficients and CI95% in Lyon and Le Bugey with and without taking into account wind speed. This table shows that taking into account winds speed, decrease performance of the GIS metric. (DOCX 12 kb)

Additional file 5: Weighted kappa coefficients and CI95% in Lyon, Le Bugey and Le Havre with different source technical parameter settings. This table shows the variation of the concordance between the two classifications according to the combination of the setting of sources technical parameters (stack height and smoke velocity) in Lyon, Le Bugey and Le Havre scenarios. (DOCX 13 kb)

Additional file 6: Weighted kappa coefficients and CI95% in Lyon, Le Bugey and Le Havre with and without technical parameters. This table shows the variation of the concordance between the two classifications across the 3 areas (Le Havre, Lyon and Le Bugey) with and without taking account sources technical parameters (stack height and smoke velocity). (DOCX 14 kb)
