# Supplementary material for: Development and performance evaluation of a GIS-based metric to assess exposure to airborne pollutant emissions from industrial sources
Source: Environ Health. 2019 Jan 25;18:8. doi: 10.1186/s12940-019-0446-x (PMC6347831; doi:10.1186/s12940-019-0446-x)
Supplement: Supplementary file 1 — Boxplot of the average dioxin concentrations (fg-TEQ/m3), modeled at the E3N location in Lyon, Le Havre and Le Bugey for 1996, 2002 and 2008 with the SIRANE model. This figure shows the reparation of subjects’ exposure to dioxin, obtained with the SIRANE model, for 3 years (1996, 2002 and 2008) for the 3 areas (Le Havre, le Bugey, Lyon). (DOCX 582 kb) [file 12940_2019_446_MOESM1_ESM.docx]

Figure S1 – Boxplot of the average dioxin concentration (fg-TEQ/m3), modeled at the E3N location in Lyon, Le Havre and Le Bugey for 1996, 2002 and 2008 with the SIRANE model

**
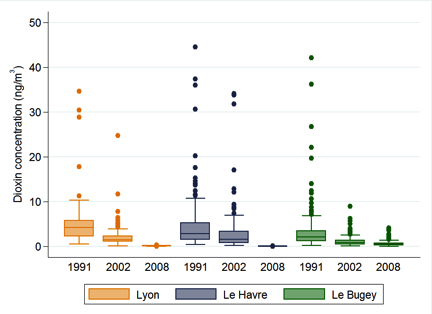
**

**Dioxin concentration (fg-TEQ/m^3^)**

The highest median dioxin concentrations (fg-TEQ/m3) were observed in 1996 in the three areas (median (min-max): Lyon 4.8 (0.6-34.7); Le Havre 2.87 (0.46- 5.7); Le Bugey 2.18 (0.25-42.2)). From 1996 to 2008, annual average dioxin concentrations decreased by 98%, 99% and 80% for Lyon, Le Bugey and Le Havre, respectively. Over the same period, we observed a relevant change in the spatial concentration patterns with the virtual extinction of the local concentration peaks due to industrial sources. As a result, the concentration field appears more homogeneous in 2008 compared to the previous years. In 2008, dioxin concentrations were lower than in 1996 and 2002 with median concentrations less than 1 fg-TEQ/m3 (median (min-max): Lyon 0.08 (0.005-0.34); Le Havre 0.02 (0.003- 0.15); Le Bugey 0.51 (0.51-4.19)).
