# Supplementary material for: Development and performance evaluation of a GIS-based metric to assess exposure to airborne pollutant emissions from industrial sources
Source: Environ Health. 2019 Jan 25;18:8. doi: 10.1186/s12940-019-0446-x (PMC6347831; doi:10.1186/s12940-019-0446-x)
Supplement: Supplementary file 2 — Boxplot of the average cadmium concentrations (ng/m3), modeled at the E3N location in Lyon, Le Havre and Le Bugey for 1996, 2002 and 2008 with the SIRANE model. This figure shows the reparation of subjects’ exposure to cadmium, obtained with the SIRANE model, for 3 years (1996, 2002 and 2008) for the 3 areas (Le Havre, le Bugey, Lyon). (DOCX 737 kb) [file 12940_2019_446_MOESM2_ESM.docx]

Additional file 2 - Boxplot of the average cadmium concentration (ng/m^3^), modeled at the E3N location in Lyon, Le Havre and Le Bugey for 1996, 2002 and 2008 with the SIRANE model


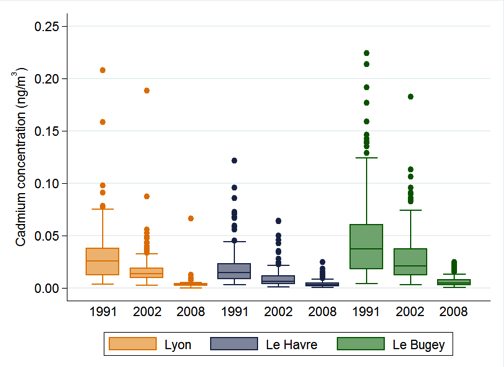


.

The highest median concentrations were observed in 1996 (median (min-max) in ng/m3: Lyon 0.032 (0.001-0.378); Havre 0.015 (0.003- 0.649); Bugey 0.039 (0.004-0.744)). We also observed a decrease in cadmium concentration over the 1996-2008 periods (by 89%, 86% and 79% for Lyon, Le Bugey and Le Havre, respectively). In 2008, cadmium concentrations were less than 0.05 fg-TEQ/m3 (median (min-max): Lyon 0.003 (0.0003-0.008); Havre 0.003 (0.001- 0.025); Bugey 0.005 (0.001-0.082)). This change in cadmium concentration is due to the decrease in emissions. Cadmium concentration field was more homogeneous and subjects were exposed to lower cadmium concentrations in 1996 than in 2002 and 2008.
