# Supplementary material for: Development and performance evaluation of a GIS-based metric to assess exposure to airborne pollutant emissions from industrial sources
Source: Environ Health. 2019 Jan 25;18:8. doi: 10.1186/s12940-019-0446-x (PMC6347831; doi:10.1186/s12940-019-0446-x)
Supplement: Supplementary file 3 — Weighted kappa coefficients and CI95% with different CADD and distance decline patterns in the Le Bugey scenarios. This table shows the variation of the concordance between the two classifications according to the combination of the setting of different parameters (winds direction and distance decline) in Le Bugey scenario. (DOCX 14 kb) [file 12940_2019_446_MOESM3_ESM.docx]

Additional file 3 - Weighted kappa coefficients and CI95 % with different CADD and distance decline patterns in Le Bugey scenarios.

| Distance decline | Year | Without integration of wind direction | 90°; 4 CADD | 30°; 12 CADD | 10°; 36 CADD | 10° with adjacent segments; weighted at 25% | 10° with adjacent segments; weighted at 50% |
| --- | --- | --- | --- | --- | --- | --- | --- |
| 1/d | 1996 | 0.64 (0.56-0.72) | 0.58 (0.50-0.67) | 0.63 (0.55-0.70) | 0.65 (0.58-0.72) | 0.66 (0.59-0.73) | 0.63 (0.55-0.70) |
|  | 2002 | 0.78 (0.73-0.84) | 0.58 (0.51-0.66) | 0.59 (0.51-0.70) | 0.58 (0.50-0.67) | 0.58 (0.49-0.66) | 0.58 (0.50-0.65) |
|  | 2008 | 0.68 (0.60-0.75) | 0.51 (0.42-0.60) | 0.53 (0.44-0.63) | 0.53 (0.45-0.62) | 0.53 (0.44-0.61) | 0.52 (0.43-0.61) |
| 1/d² | 1996 | 0.59 (0.51-0.68) | 0.67 (0.59-0.74) | 0.73 (0.67-0.80) | 0.75 (0.69-0.81) | 0.76 (0.71-0.84) | 0.79 (0.73-0.85) |
|  | 2002 | 0.74 (0.68-0.80) | 0.78 (0.72-0.83) | 0.84 (0.79-0.89) | 0.83 (0.77-0.88) | 0.83 (0.78-0.88) | 0.82 (0.76-0.87) |
|  | 2008 | 0.64 (0.57-0.72) | 0.67 (0.60-0.74) | 0.72 (0.65-0.78) | 0.73 (0.67-0.80) | 0.74 (0.68-0.80) | 0.73 (0.66-0.79) |
| e^-d^ | 1996 | 0.50 (0.40-0.60) | 0.56 (0.47-0.65) | 0.63 (0.54-0.71) | 0.64 (0.56-0.72) | 0.65 (0.57-0.73) | 0.65 (0.57-0.73) |
|  | 2002 | 0.65 (0.58-0.72) | 0.73 (0.67-0.80) | 0.77 (0.71-0.83) | 0.78 (0.72-0.84) | 0.78 (0.72-0.84) | 0.78 (0.72-0.84) |
|  | 2008 | 0.58 (0.50-0.66) | 0.67 (0.60-0.74) | 0.66 (0.59-0.73) | 0.66 (0.59-0.73) | 0.67 (0.60-0.74) | 0.67 (0.60-0.74) |
| e^-d^/d | 1996 | 0.45 (0.35-0.55) | 0.49 (0.39-0.59) | 0.51 (0.42-0.60) | 0.54 (0.45-0.63) | 0.53 (0.44-0.61) | 0.53 (0.44-0.61) |
|  | 2002 | 0.63 (0.56-0.71) | 0.66 (0.58-0.73) | 0.73 (0.66-0.79) | 0.74 (0.67-0.81) | 0.73 (0.66-0.80) | 0.73 (0.67-0.80) |
|  | 2008 | 0.57 (0.48-0.65) | 0.60 (0.52-0.68) | 0.59 (0.51-0.67) | 0.62 (0.54-0.69) | 0.62 (0.54-0.69) | 0.62 (0.54-0.69) |
| 1/d^1,5^ | 1996 | 0.61 (0.53-0.69) | 0.66 (0.58-0.74) | 0.73 (0.67-0.80) | 0.72 (0.65-0.78) | 0.73 (0.67-0.80) | 0.73 (0.67-0.80) |
|  | 2002 | 0.78 (0.72-0.83) | 0.69 (0.63-0.75) | 0.78 (0.73-0.84) | 0.74 (0.68-0.80) | 0.76 (0.70-0.82) | 0.74 (0.68-0.80) |
|  | 2008 | 0.68 (0.61-0.76) | 0.62 (0.54-0.69) | 0.64 (0.56-0.72) | 0.66 (0.58-0.73) | 0.66 (0.58-0.73) | 0.65 (0.58-0.72) |
| 1/d^3^ | 1996 | 0.51 (0.42-0.60) | 0.58 (0.49-0.66) | 0.69 (0.62-0.77) | 0.70 (0.63-0.77) | 0.72 (0.64-0.79) | 0.72 (0.65-0.79) |
|  | 2002 | 0.68 (0.61-0.76) | 0.78 (0.71-0.84) | 0.83 (0.77-0.88) | 0.83 (0.78-0.89) | 0.85 (0.80-0.90) | 0.85 (0.80-0.90) |
|  | 2008 | 0.62 (0.54-0.69) | 0.68 (0.61-0.75) | 0.68 (0.61-0.74) | 0.67 (0.60-0.73) | 0.69 (0.62-0.76) | 0.69 (0.62-0.76) |
| e^-d^*d | 1996 | 0.53 (0.44-0.63) | 0.66 (0.60-0.75) | 0.70 (0.63-0.77) | 0.75 (0.68-0.82) | 0.75 (0.68-0.82) | 0.75 (0.68-0.82) |
|  | 2002 | 0.65 (0.58-0.72) | 0.74 (0.68-0.80) | 0.78 (0.73-0.84) | 0.80 (0.75-0.85) | 0.82 (0.77-0.87) | 0.81 (0.76-0.86) |
|  | 2008 | 0.60 (0.52-0.68) | 0.64 (0.57-0.71) | 0.68 (0.62-0.75) | 0.68 (0.62-0.75) | 0.68 (0.62-0.75) | 0.68 (0.62-0.75) |
