# Supplementary material for: Development and performance evaluation of a GIS-based metric to assess exposure to airborne pollutant emissions from industrial sources
Source: Environ Health. 2019 Jan 25;18:8. doi: 10.1186/s12940-019-0446-x (PMC6347831; doi:10.1186/s12940-019-0446-x)
Supplement: Supplementary file 4 — Weighted kappa coefficients and CI95% in Lyon and Le Bugey with and without taking into account wind speed. This table shows that taking into account winds speed, decrease performance of the GIS metric. (DOCX 12 kb) [file 12940_2019_446_MOESM4_ESM.docx]

Additional file 4 - Weighted kappa coefficients and CI95 % in Lyon and Le Bugey with and without taking into account wind speed.

| Area | Year | Without wind speed | With wind speed |
| --- | --- | --- | --- |
| Le Bugey | 1996 | 0.79 (0.73-0.85) | 0.76 (0.70-0.82) |
|  | 2002 | 0.82 (0.76-0.87) | 0.58 (0.49-0.66) |
|  | 2008 | 0.73 (0.66-0.79) | 0.59 (0.51-0.67) |
| Lyon | 1996 | 0.71 (0.67-0.76) | 0.68 (0.63-0.73) |
|  | 2002 | 0.84 (0.79-0.88) | 0.59 (0.52-0.66) |
|  | 2008 | 0.81 (0.72-0.89) | 0.71 (0.61-0.81) |
