# Supplementary material for: Development and performance evaluation of a GIS-based metric to assess exposure to airborne pollutant emissions from industrial sources
Source: Environ Health. 2019 Jan 25;18:8. doi: 10.1186/s12940-019-0446-x (PMC6347831; doi:10.1186/s12940-019-0446-x)
Supplement: Supplementary file 5 — Weighted kappa coefficients and CI95% in Lyon, Le Bugey and Le Havre with different source technical parameter settings. This table shows the variation of the concordance between the two classifications according to the combination of the setting of sources technical parameters (stack height and smoke velocity) in Lyon, Le Bugey and Le Havre scenarios. (DOCX 13 kb) [file 12940_2019_446_MOESM5_ESM.docx]

Additional file 5 - Weighted kappa coefficients and CI95 % in Lyon, Le Bugey and Le Havre with different source technical parameter settings

| Parameters | Years | Lyon | Le Bugey | Havre |
| --- | --- | --- | --- | --- |
|  | 1996 | 0.64 (0.59-0.69) | 0.72 (0.66 -0.79) | 0.70 (0.63-0.77) |
| $\frac{1}{h}$ | 2002 | 0.77 (0.70-0.83) | 0.77 (0.71-0.82) | 0.78 (0.72-0.84) |
|  | 2008 | 0.75 (0.64-0.86) | 0.77 (0.71-0.83) | 0.73 (0.66-0.79) |
|  | 1996 | 0.68 (0.64-0.73) | 0.72 (0.66-0.79) | 0.73 (0.66-0.79) |
| $\frac{1}{\sqrt{h}}$ | 2002 | 0.81 (0.77-0.86) | 0.77 (0.72-0.83) | 0.80 (0.74-0.86) |
|  | 2008 | 0.79 (0.70-0.88) | 0.73 (0.66-0.79) | 0.76 (0.70-0.82) |
|  | 1996 | 0.57 (0.51-0.64) | 0.71 (0.65-0.78) | 0.55 (0.46-0.64) |
| $\frac{1}{h^{2}}$ | 2002 | 0.70 (0.62-0.78) | 0.77 (0.71-0.83) | 0.64 (0.56-0.72) |
|  | 2008 | 0.56 (0.42-0.70) | 0.69 (0.63-0.76) | 0.61 (0.53-0.69) |
|  | 1996 | 0.67 (0.62-0.71) | 0.71 (0.65-0.78) | 0.71 (0.64-0.78) |
| $\frac{1}{h*v}$ | 2002 | 0.78 (0.72-0.84) | 0.77 (0.71-0.83) | 0.81 (0.75-0.86) |
|  | 2008 | 0.81 (0.72-0.90) | 0.79 (0.73-0.85) | 0.73 (0.66-0.79) |
|  | 1996 | 0.70 (0.66-0.75) | 0.71 (0.65-0.78) | 0.77 (0.71-0.83) |
| $\frac{1}{\sqrt{h}*v}$ | 2002 | 0.82 (0.77-0.87) | 0.76 (0.70-0.82) | 0.81 (0.75-0.86) |
|  | 2008 | 0.81 (0.73-0.89) | 0.77 (0.71-0.83) | 0.73 (0.67-0.80) |
|  | 1996 | 0.60 (0.54-0.66) | 0.70 (0.64-0.77) | 0.57 (0.48-0.65) |
| $\frac{1}{h^{2}*v}$ | 2002 | 0.71 (0.63-0.78) | 0.77 (0.71-0.83) | 0.70 (0.63-0.77) |
|  | 2008 | 0.66 (0.53-0.78) | 0.69 (0.62-0.75) | 0.63 (0.56-0.71) |
