# Supplementary material for: Development and performance evaluation of a GIS-based metric to assess exposure to airborne pollutant emissions from industrial sources
Source: Environ Health. 2019 Jan 25;18:8. doi: 10.1186/s12940-019-0446-x (PMC6347831; doi:10.1186/s12940-019-0446-x)
Supplement: Supplementary file 6 — Weighted kappa coefficients and CI95% in Lyon, Le Bugey and Le Havre with and without technical parameters. This table shows the variation of the concordance between the two classifications across the 3 areas (Le Havre, Lyon and Le Bugey) with and without taking account sources technical parameters (stack height and smoke velocity). (DOCX 14 kb) [file 12940_2019_446_MOESM6_ESM.docx]

Additional file 6 - Weighted kappa coefficients and CI95 % in Lyon, Le Bugey and Le Havre with and without technical parameters

| Areas | Years | $GIS metric \left( {g-TEQ.m}^{-2} \right)$  $=\sum_{j}^{J} \sum_{i}^{I} t\times\frac{1}{{d^{2}}_{i}}\times\mathrm{EI}_{i}\times F_{i}$ | $GIS metric \left( {g-TEQ.m}^{-2} \right)$  $=\sum_{j}^{J} \sum_{i}^{I} t\times\frac{1}{{d^{2}}_{i}}\times\mathrm{EI}_{i}\times F_{i}\times\frac{1}{\sqrt{h}*v}$ |
| --- | --- | --- | --- |
| Lyon | 1996 | 0.71 (0.67-0.76) | 0,70 (0,66-0,75) |
|  | 2002 | 0.84 (0.79-0.88) | 0,82 (0,77-0,87) |
|  | 2008 | 0.81 (0.72-0.89) | 0,81 (0,73-0,89) |
| Le Bugey | 1996 | 0.79 (0.73-0.85) | 0,71 (0,65-0,78) |
|  | 2002 | 0.82 (0.76-0.87) | 0,76 (0,70-0,82) |
|  | 2008 | 0.73 (0.66-0.79) | 0,77 (0,71-0,83) |
| Le Havre | 1996 | 0,74 (0,68-0,80) | 0,77 (0,71-0,83) |
|  | 2002 | 0,77 (0,72-0,83) | 0,81 (0,75-0,86) |
|  | 2008 | 0,64 (0,57-0.72) | 0,73 (0,67-0,80) |

We observed a clear improvement of the results for the scenario Le Havre 2008. In this specific scenario, the major source had a stack height of 240m that impacted dioxin dispersion and decrease dioxin exposure. As explained in 3.1, given the absence of impact of the stack height on the kappa coefficienst in all other scenarios and the low completeness of data for stack height at the national level (36%), it was decided to integrate into the GIS metric only stacks above 90m, corresponding to 3 times the median stack height of the 2,626 sources at the national level. This led us to the final GIS metric:

$$GIS-based metric \left( {g-TEQ.m}^{-2} \right)=\sum_{j}^{J} \sum_{i}^{I} t\times\frac{1}{{d^{2}}_{i}}\times\mathrm{EI}_{i}\times F_{i}\times\left( \frac{h_{\mathrm{median}}}{h_{i}} \right)^{*} (i)$$
